# Supplementary figures and images for: Independent regulation of Z-lines and M-lines during sarcomere assembly in cardiac myocytes revealed by the automatic image analysis software sarcApp
Source: eLife. 2023 Nov 3;12:RP87065. doi: 10.7554/eLife.87065 (PMC10624428; doi:10.7554/eLife.87065)

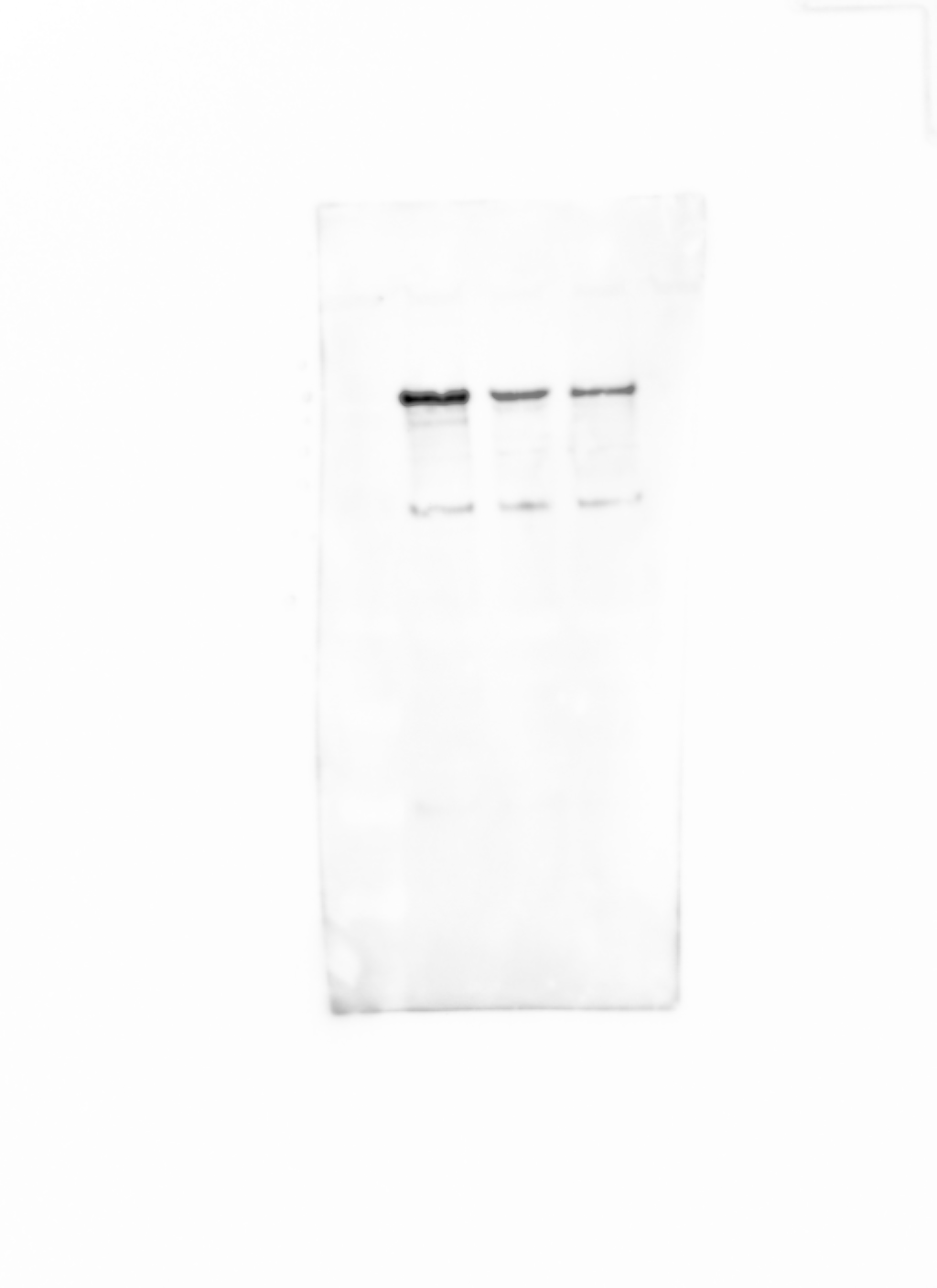

Supplement: Figure 7—source data 1. — See Figure 7-figure supplement 7A and Figure 7B. [file elife-87065-fig7-data1.zip › Figure 7-source data 1 Figure 7B siMYH6 anti MYH6.tif]

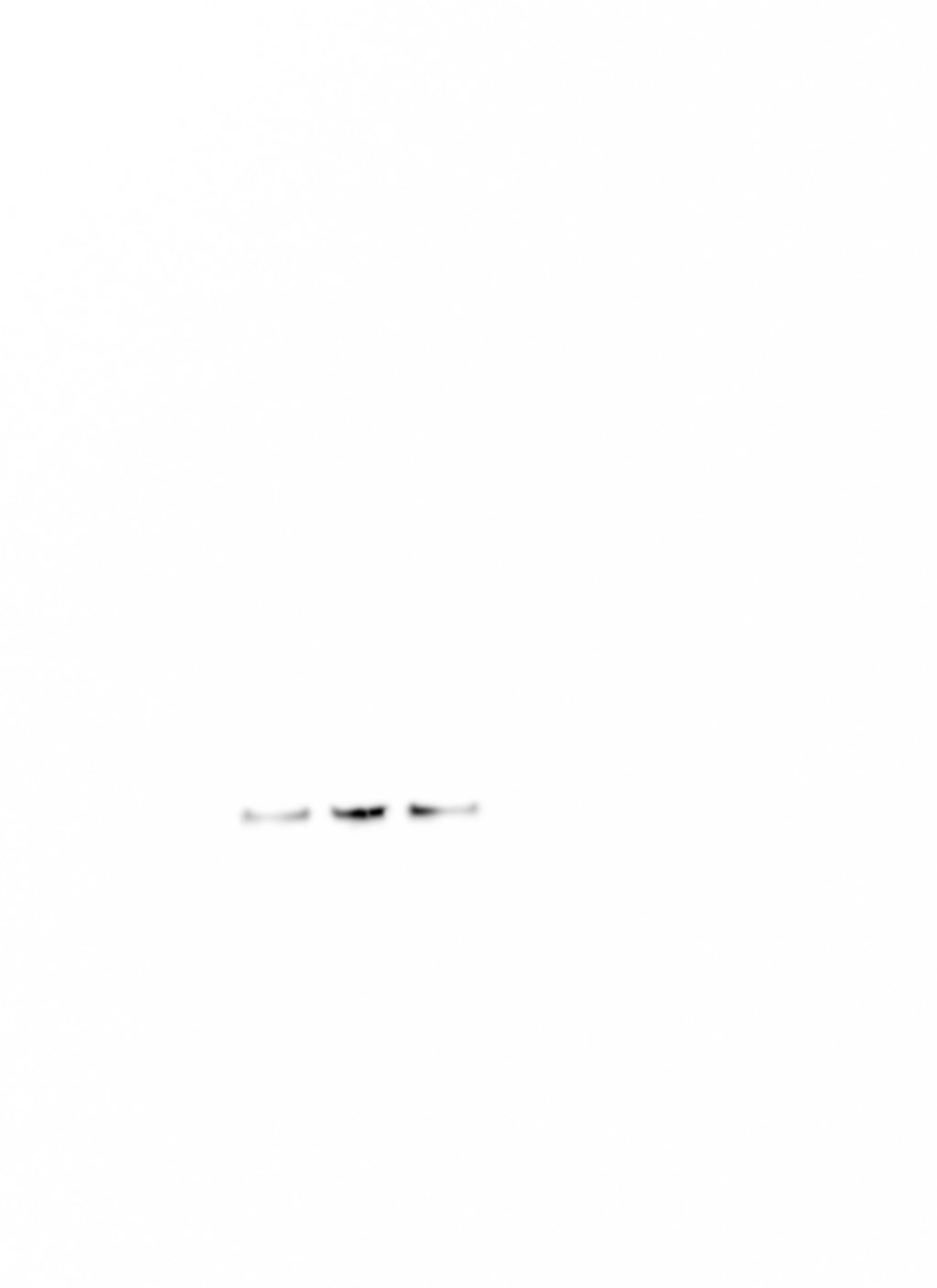

Supplement: Figure 7—source data 2. — See Figure 7-figure supplement 7A and Figure 7B. [file elife-87065-fig7-data2.zip › Figure 7-source data 2 Figure 7B siMYH6 anti tubuin.tif]

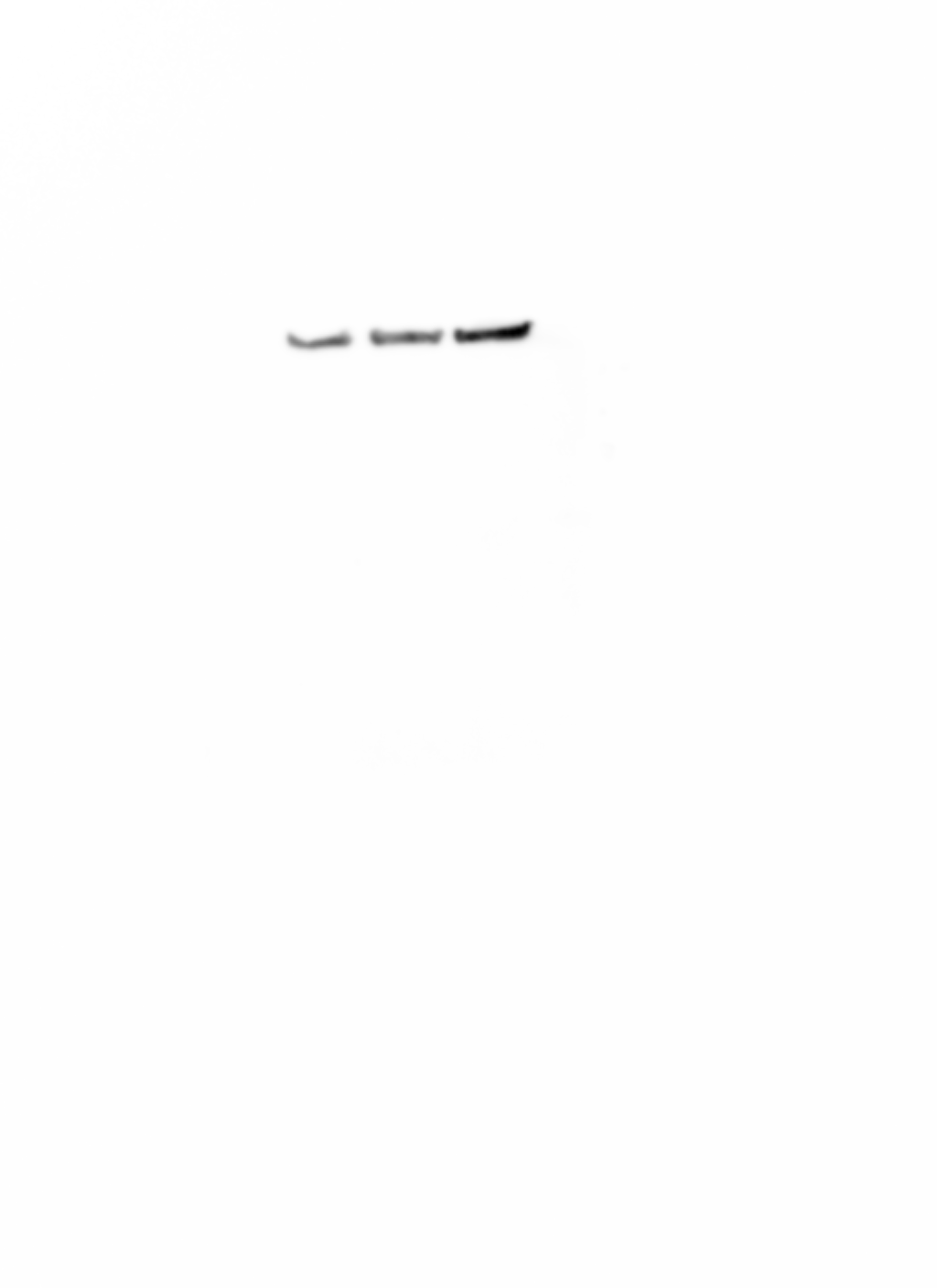

Supplement: Figure 7—source data 3. — See Figure 7-figure supplement 7B and Figure 7H. [file elife-87065-fig7-data3.zip › Figure 7-source data 3 Figure 7H siMYH7 anit tubulin.tif]

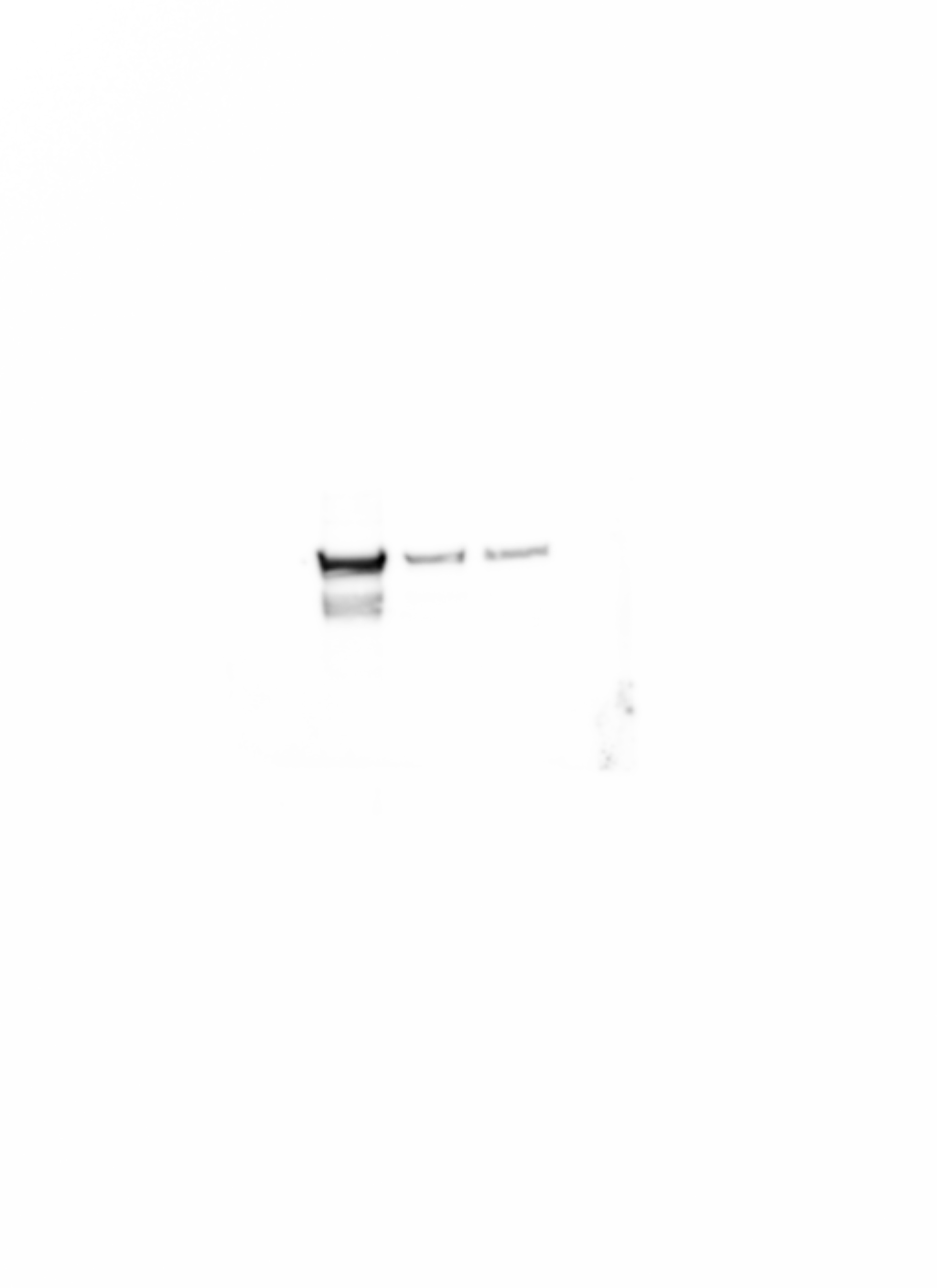

Supplement: Figure 7—source data 4. — See Figure 7-figure supplement 7B and Figure 7H. [file elife-87065-fig7-data4.zip › Figure 7-source data 4 Figure 7H siMYH7 anti MYH7.tif]

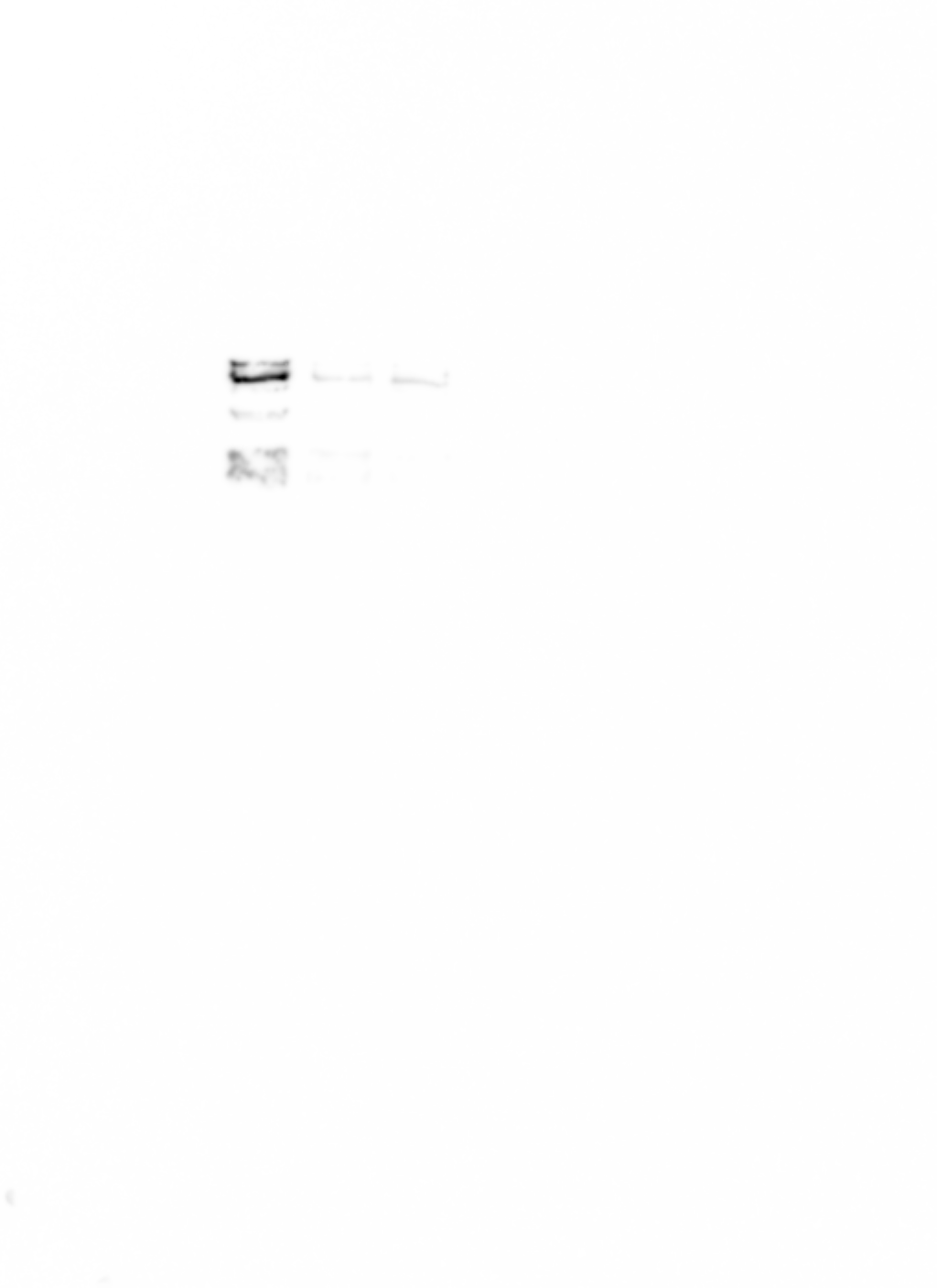

Supplement: Figure 8—source data 1. — See Figure 8—figure supplement 3A and Figure 8B. [file elife-87065-fig8-data1.zip › Figure 8-source data 1 Figure 8B siMYOM anti MYOM.tif]

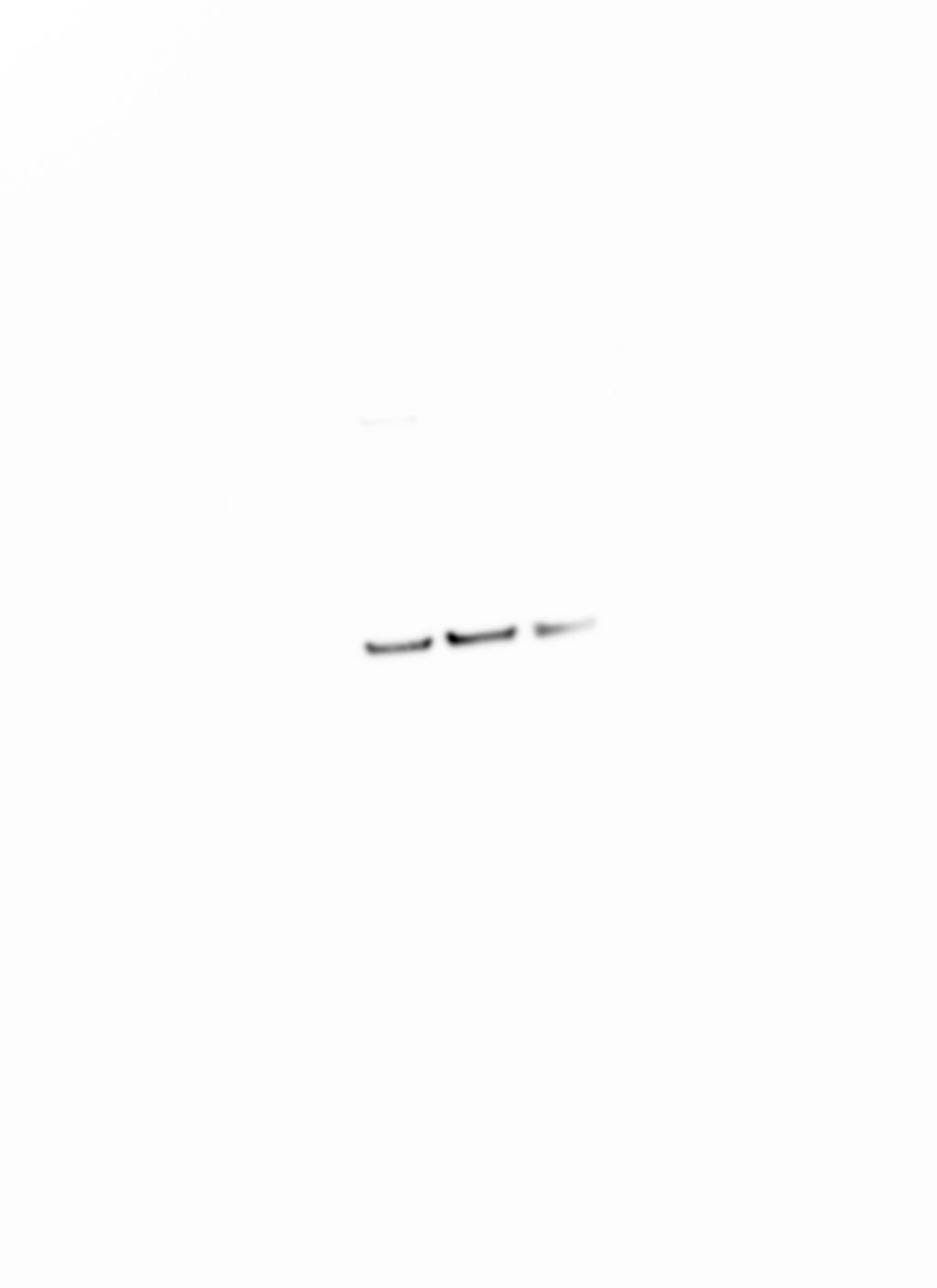

Supplement: Figure 8—source data 2. — See Figure 8—figure supplement 3A and Figure 8B. [file elife-87065-fig8-data2.zip › Figure 8-source data 2 Figure 8B siMYOM anti tubulin.tif]
